# Supplementary material for: Psychological impact, coping behaviors, and traumatic stress among healthcare workers during COVID-19 in Taiwan: An early stage experience
Source: PLoS One. 2022 Oct 25;17(10):e0276535. doi: 10.1371/journal.pone.0276535 (PMC9595532; doi:10.1371/journal.pone.0276535)
Supplement: Attachment — Submitted filename: Response to Reviewers.docx [file pone.0276535.s002.docx]

Dear reviewer

Thank you for your kind review and constructive recommendations. Our reply is as follows:

**General comments**

**The title of the manuscript should be including the study setting and design. The word “Low-Disease-Burden Country” seems to be a general term**

Answer：Thanks for your comment.

In order to make the setting of the study more clear to readers, we decided to modify the title to "Psychological Impact, Coping Behaviors, and Traumatic Stress among Healthcare Workers during COVID-19 in Taiwan: An Early Stage Experience". And describe that the impact of the pandemic during this period was significantly lower in Taiwan than in other countries in the introduction.

**Introduction**

**The statement “given that Taiwan was one of the worst-hit territories in the severe acute respiratory syndrome (SARS) outbreak of 2003……..” needs a reference.**

Answer：Thanks for your comment and reminder.

We mark cited references and revise the numbering of the references.(Line 56)

**Methods**

**Please include the sample size estimation for this study.**

**The sampling method should be mentioned.**

**How was the data collected from the respondents?**

Answer：Thanks for your comment.

The study subjects included all HCWs employed by the study hospitals, as there were no sampling issues to consider. The process of data collection was added to the text.(Line105-108)

**Results**

**Line 173: Please check the percentage of the respondents in table 1. The total should be 100% for variables, and some variables have got more than 100% (eg, age), and some got less than 100% (eg, education level).**

Answer：Thanks for your comment.

These results are due to rounding. After reviewing the raw data again, some data are corrected to match the total to 100%.(Line 159, Table 1)

**Line 173: In demographic variables, “living with …..” has 155.1%. Did the multiple answers allow for that variable? If yes, pls indicate in the table footnote.**

Answer：Thanks for your comment.

Answer yes or no to each question as indicated in the footnotes. (Line 196)

**The majority of the respondents were nurses (84.5%) and 15.5% were physicians. Is there any pre-determined ratio depending on the occupation?**

Answer：Thanks for your comment.

The study hospital employed 64 (16.9%) physicians and 314 nurses (83.1%)(in Line 113), which was not significantly different from the rate of 55 (15.5%) responding physicians and 299 nurse practitioners (84.5%).

**Line 285: “The respondents had a mean IES-R score of 17.01 ± 14.16 (0–74).” It would be better if the authors mentioned about mean, SD, minimum – maximum in the sentence.**

Answer：Thanks for your comment.

We remove mean so that this sentence represents all data about IES-R.(Line 307)

**Discussion**

**Line 332: The first paragraph of the discussion focused on the context of the study, the COVID-19 situation, and responses in Taiwan. This paragraph is more suitable in the introduction rather than the discussion. The discussion should start with the overall major findings of the study and compare the similarities and differences with previous literature.**

Answer：Thanks for your comment.

We moved the text of the first paragraph to the introduction, as suggested by the reviewers.(Line77-91)

**The authors may aware that many studies have been investigated the impact of COVID-19 on mental health, coping strategies, and traumatic stress. I would like to suggest the authors to highlight the unique findings of your study and mention them in the discussion.**

Answer：Thanks for your comment.

We partially revised the original first paragraph to show that although the pandemic was less severe, the effects of perceived impact and coping behaviors were moderate.(Line 354-355)

**Limitations**

**What was the sampling method to recruit the respondents? Is that method affect on the** **generalizability of the findings?**

Answer：Thanks for your comment.

Because all HCWs in the study hospitals were included in the study subjects, there was no sampling issue in this study. Generalizability of limitations will still be concentrated in a single study setting that had described in research limitations (in Line 463).

The authors once again thank the reviewers for reading this manuscript in detail and for making constructive recommendations.
